# Mammary Paget's disease occurring after mastectomy

**DOI:** 10.1186/1477-7819-4-51

**Published:** 2006-08-09

**Authors:** Monica Giovannini, Carmelo D'Atri, Quirino Piubello, Annamaria Molino

**Affiliations:** 1 Department of Medical Oncology, San Raffaele Scientific Institute, Milan, Italy; 2 Department of Surgery, Civic Hospital, Verona, Italy; 3 Department of Pathology, Civic Hospital, Verona, Italy; 4 Department of Medical Oncology, University of Verona, Italy

## Abstract

**Background:**

Mammary Paget's disease and extramammary Paget's disease are neoplastic conditions, in which there is intraepithelial (usually intraepidermal) infiltration by neoplastic cells showing glandular differentiation. Mammary Paget's disease occurs exclusively on the nipple/areola complex from where it may spread to the surrounding skin.

**Case presentation:**

We here describe a case of Paget's disease occurring on the thoracic wall site of a previous simple mastectomy, and also briefly summarise the most important aspects leading to a diagnosis of mammary Paget's disease.

**Conclusion:**

To the best of our knowledge, this is the first reported case of mammary Paget's disease occurring after mastectomy. The absence of the nipple/areola complex obviously raised some questions concerning whether it was mammary or extra-mammary Paget's disease, and how it could occur in the absence of the nipple/areola complex.

## Background

Mammary Paget's disease occurs exclusively on the nipple/areola complex from where it may spread on to surrounding skin. Extramammary Paget's disease occurs most commonly in the anogenital region but can arise in any area of skin and mucosa.

Mammary Paget's disease accounts for 2–3% of neoplastic conditions of the breast and in most cases (82–92% in several studies) tumour cells have spread to the skin of the nipple and areola from underlying invasive carcinoma or ductal carcinoma in situ [1-3]. The neoplasm associated with mammary Paget's disease, which may or may not be palpable, is usually centrally located (within 2 cm of the areola) but occasionally may be more peripherally sited [1,2]. In cases where a mass is palpable, invasive carcinoma is likely to be found. Conversely, cases of mammary Paget's disease with no palpable mass are more likely to have ductal carcinoma in situ only (66% of cases in one study) [2].

Mammary Paget's disease has been reported in the male breast with no evidence that the disease behaves differently, although the numbers of cases reported are small [4].

This paper describes a case of Paget's disease occurred on the thoracic wall where simple mastectomy was performed many years before.

## Case presentation

A 57-year-old woman underwent simple mastectomy (according to Madden procedure) and axillary dissection on 7 December 1995. She reported the appearance of eczema of the nipple-areola complex some years before, and the presence of a palpable mass for about two months; no mention was made of nipple discharge or ulceration. Histological examination revealed unifocal Paget's disease of the nipple underlying an invasive mucinous ductal breast carcinoma (pT2 2.2 cm) with focal aspects of intraductal carcinoma; none of the 21 nodes obtained upon axillary dissection were involved in the disease. Immunohistochemically, the tumour presented high estrogen and low progesterone receptor positivity, and a low-intermediate proliferative rate (Ki67 14%). No secondary lung, liver or bone lesions were detected. On the basis of the disease stage and histology, and age at diagnosis, adjuvant hormonal treatment with tamoxifen was started on 9 January 1996.

In February 1999, the patient complained of a 1 × 1.5 cm hyperpigmented eczematoid erythematous area at the site of ths previous mastectomy, whose size remained unchanged for four years.

**Figure 1:**
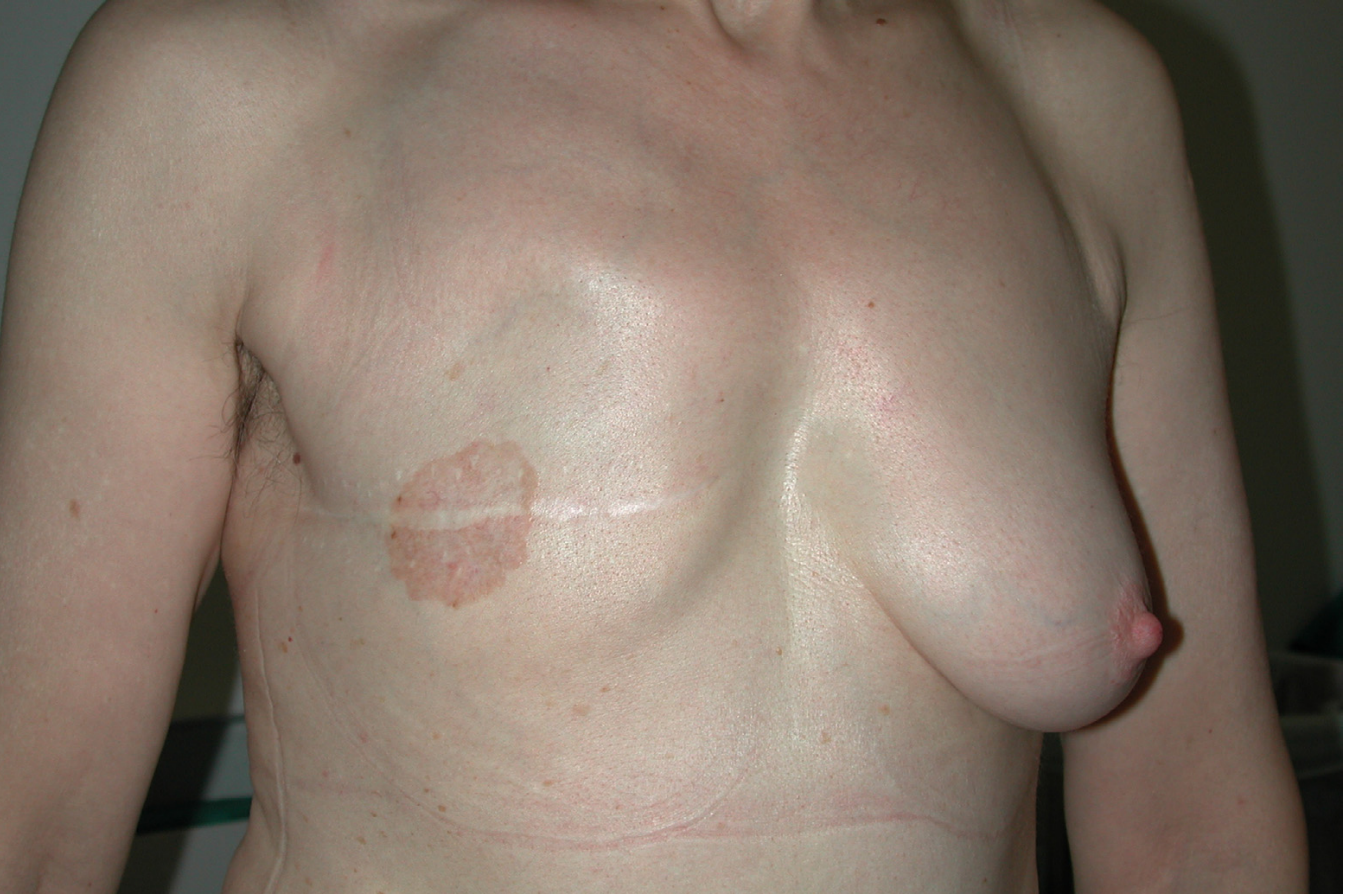
Clinical aspects of mammary Paget's disease occurred on the thoracic wall where simple mastectomy was performed some years before.

**Figure 2:**
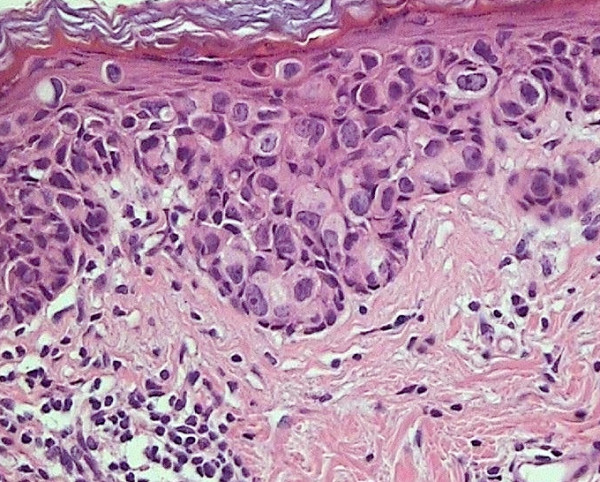
Histological examination of Page's disease: Paget's cells, with abundant pale cytoplasmand pleomorphic nuclei, replace the basal epidermis and are scattered individually throughout the squamous epithelium.

The beginning of its slow growth in 2003 led to a dermatological evaluation being advised, but this was delayed by the patient until December 2004, when a biopsy was performed. No scraping was possible as there was no crusted lesion (Figure 1). Histological examination showed Page's disease (immunohistochemistry: ER 30%+, PgR 5%+, Ki67 19%+, c-erbB2 positive, CEA and EMA positive; cytokeratin 20 and 5 negative, cytokeratine 7 and 8/18/19 positive; S100 negative). Unfortunately, the patient did not visit our clinic until three months after the diagnosis and so, on the basis of the histological diagnosis, the area was surgically resected on 22 March 2005. Histological examination confirmed the lesion to be Paget's disease of the thoracic skin; the surgical margins were negative (Figure 2). A new hormonal therapy with letrozole was subsequently started.

## Discussion

To the best of our knowledge, this is the first reported case of mammary Paget's disease occurring after mastectomy. The absence of the nipple/areola complex obviously raised some questions concerning whether it was mammary or extra-mammary Paget's disease, and how it could occur in the absence of the nipple/areola complex.

The first question was answered by the immunohistochemical examination because hormonal receptors can only be detected in mammary Paget's disease. The second one could be explained by the high prevalence of multifocal disease in patients with Paget's mammary disease. However in this case the pathologist revealed unifocal Paget's disease of the nipple underlying an invasive mucinous ductal breast carcinoma with focal aspects of intraductal carcinoma and surgical negative margins in the histological specimen in 1995 when mastectomy was performed and unifocal Paget's disease of the thoracic skin with no microinvasion and negative margins at the time of the second resection.

In order to understand this better, we here briefly summarise the most important aspects leading to the diagnosis of mammary Paget's disease, which was first described by James Paget in 1874 [5] as a neoplastic condition that exclusively affects the nipple/areola complex from where it may spread to the surrounding skin. It is clinically characterised by a demarcated, thickened, eczematoid erythematous weeping or crusted lesion with irregular borders; nipple discharge and ulceration may sometimes occur, and there may be an associated palpable breast tumour. Some cases of mammary Paget's disease are clinically occult and only detected histologically when a representative section of the nipple and areola is submitted from a mastectomy. The differential clinical diagnoses include generalised inflammatory skin conditions such as eczema and psoriasis, as well as erosive adenomatosis, a condition that is specific to the nipple.

Mammary Paget's disease accounts for 2–3% of all neoplastic conditions of the breast and, in most cases (up to 90%), microscopic examination shows intraepithelial (usually intraepidermal) infiltration by neoplastic cells with glandular differentiation. Most cases originate from *in situ *or invasive ductal carcinoma in the underlying breast tissue [1-3], but rare cases appear to have originated primarily within the nipple epidermis. The neoplasm associated with mammary Paget's disease is usually located centrally (within 2 cm of the areola) but may occasionally may be more peripheral [1,5].

When a mass is palpable, invasive carcinoma is likely to be found, which is said to be more commonly multifocal with axillary node involvement, and to lead to worse survival than in patients whose tumour does not show an epidermal spread [2]. However, other studies comparing invasive carcinoma patients with and without mammary Paget's disease have found that the most important prognostic factor is the presence or absence of axillary metastases, rather than the presence of skin involvement [6]. Conversely, patients with mammary Paget's disease and no palpable mass are more likely to have *in situ *ductal carcinoma only [2]. The breast cancer associated with mammary Paget's disease is often multifocal.

The surgical management of mammary Paget's disease associated with neoplastic disease consists of mastectomy, which is frequent in such patients because of the high prevalence of multifocal disease; breast-conserving surgery, which in these cases consists of a wide local excision of the tumour with the excision of the nipple/areola complex, is regarded as being more appropriate to an underlying unifocal breast tumour. Conservative surgery with adjuvant radiotherapy may be possible in selected cases [7,8], although radiotherapy alone has been proposed as a viable alternative to surgery for the rare cases that lack any signs of associated neoplastic disease in the underlying breast tissue [8-10].

The presence of an associated invasive tumour obviously drives the therapeutic decision-making process. Immunohistochemistry can be very helpful in diagnosing mammary Paget's disease, particularly in cases like ours with an unusual clinical appearance, because pathologists can make some immunohistochemical analysis using particular markers, some of which are quite sensitive or specific. Among the cytokeratins, cytokeratin 7 and 20 are sensitive but not specific markers of mammary Paget's disease, and most cases are labelled by EMA antibody. Only approximately 35% of the cases of mammary Paget's disease show anti-CEA antibodies. The tumour cells in mammary Paget's disease generally have a similar immunohistochemical steroid receptor profile to that of the associated breast carcinoma; c-erbB2 is detected in most cases of mammary Paget's disease (90–100%) and in the associated breast tumour in about 44% of cases of *in situ *ductal carcinoma, and is more commonly present in high-grade (comedo and micropapillary) lesions. S100 is useful in differentiating Paget's disease from melanoma, but may be expressed in up to 25% of cases of mammary Paget's disease (a similar frequency to that observed in mammary carcinoma) [11].

## Conflict of interests

The author(s) declare that they have no competing interests.

## Authors' contributions

**MG **as an oncologist observed for the first time the lesion on the thoracic wall and described the case, reviewing the existing literature on this topic.

**CD **as a surgeon performed the surgical resection of the lesion and took the pictures before its removal.

**QP **as a pathologist performed the histological examination of the lesion.

**AM **as an oncologist worked as the supervisor of the breast team involved in this case.

All authors read and approved the manuscript.
